# The relationship between spindly leg syndrome incidence and water composition, overfeeding, and diet in newly metamorphosed harlequin frogs (*Atelopus* spp.)

**DOI:** 10.1371/journal.pone.0204314

**Published:** 2018-10-16

**Authors:** Julio Federico Camperio Ciani, Jorge Guerrel, Eric Baitchman, Rigoberto Diaz, Matthew Evans, Roberto Ibáñez, Heidi Ross, Eric Klaphake, Bradley Nissen, Allan P. Pessier, Michael L. Power, Caitlin Arlotta, Donna Snellgrove, Brad Wilson, Brian Gratwicke

**Affiliations:** 1 Smithsonian Tropical Research Institute, Panama Amphibian Rescue and Conservation Project, Panamá; 2 Zoo New England, 1 Franklin Park Road, Boston, MA, United States of America; 3 Smithsonian’s National Zoo and Conservation Biology Institute, Washington, DC, United States of America; 4 Sistema Nacional de Investigación, Secretaría Nacional de Ciencia, Tecnología e Innovación, Panamá; 5 Cheyenne Mountain Zoo, Colorado Springs, CO, United States of America; 6 College of Veterinary Medicine, Washington State University, Pullman, WA, United States of America; 7 Waltham Center for Pet Nutrition, Freeby Lane, Waltham on the Wolds, Leicestershire, United Kingdom; 8 Atlanta Botanical Gardens, Atlanta, GA, United States of America; Universitat Trier, GERMANY

## Abstract

Spindly Leg Syndrome (SLS) is a persistent animal welfare issue associated with the rearing of amphibians in captivity. We conducted two experiments to investigate the effects of diet, water composition and overfeeding on prevalence of SLS in newly metamorphosed harlequin frogs (*Atelopus* spp.). In our first experiment, we offered 400 full-sibling tadpoles of *Atelopus certus* isocaloric diets in treatments of 31%, 37%, 42% and 48% crude protein respectively. Tadpoles fed higher protein diets metamorphosed faster, but the incidence of SLS exceeded 80% in all treatments leading to the conclusion that variation in dietary protein was not responsible for causing SLS. We used 720 full-sibling *Atelopus glyphus* tadpoles in a second experiment to examine the effects of diet type, water composition and diet ration on SLS. We found that an overall incidence of 58% spindly leg in tadpoles reared in tap water, but reduced to about 10% in water treated by reverse osmosis and then reconstituted. It is possible that the reverse osmosis treatment removed some factor that caused the SLS, or that the reconstitution may have added a mineral lacking in the original tap water. Within tap water treatments, overfeeding tadpoles in tanks increased the incidence of SLS. We recommend further experimental research into this condition to identify the causative factors in the water. Additional research into the nutritional composition of food available to wild tadpoles would be useful in formulating captive diets, that have to date been solely based on surrogate species.

## Introduction

Spindly Leg Syndrome (SLS) is a musculoskeletal abnormality commonly associated with captive-rearing of amphibian larvae resulting in under-developed front limbs [1–4]. The causes of SLS are poorly understood [2,3,5,6] some postulate a nutrient deficiency in larval diets such as vitamin B complex [3], husbandry factors such as water quality, or some complex multifactorial cause including genetics and maternal nutrition, but there has been a lack of controlled studies of the underlying etiology. The problem is a widespread and enigmatic amphibian health and welfare issue documented in several wildlife medicine text books [3,7,8].

Metabolic bone diseases lead to reduced bone density and skeletal deformities in postmetamorphic amphibians. They have been linked to improper regulation of calcium metabolism related to poor dietary Ca:P ratios, vitamin D deficiency or inadequate UV B exposure [9–14]. Calcium is a major developmental requirement during metamorphosis and tadpoles are able to selectively absorb and store calcium from their environment and is stored in endolymphatic sacs [12,15]. In amphibian tadpoles, calcium is primarily absorbed across the gills and skin, rather than the gut, and the ability to absorb calcium in this way is enhanced with dietary vitamin D supplementation [16]. Recently, others have suggested that MBD in some anurans may be a result of factors that interfere with calcium metabolism such as excessive fluoride [17], but these frogs may also have experienced conventional risk factors for MBD. Despite a significant focus on amphibian calcium metabolism in the 1970’s [12], there hasn’t been much recent attention on the subject. However, two independent studies suggest that a lack of calcium in tadpole-rearing water affected limb development [18–20]. One of these studies suggests that calcium deficiency at a key tadpole life-stage affects normal neurological development, that in turn induces limb malformations [19].

**Fig 1:**
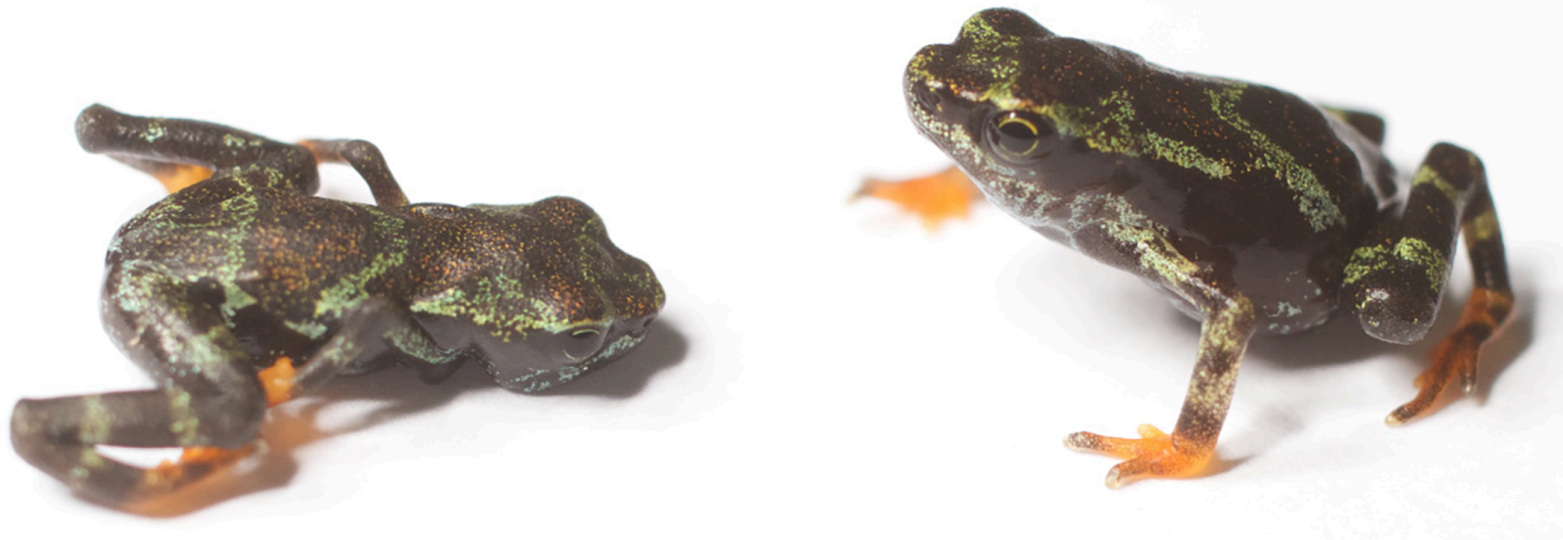
*Atelopus certus* post-metamorphs, an example of a SLS frog with poorly developed forelimbs (left) compared with a healthy froglet from the same clutch (right).

The Panama Amphibian Rescue and Conservation (PARC) Project was established in 2009 to create ex-situ assurance populations of amphibians in Panama at risk of extinction from the amphibian chytrid fungus [21]. Project staff have encountered spindly-leg syndrome in captive offspring of species with aquatic tadpoles from a variety of species including *Atelopus limosus*, *A*. *certus*, *A*. *glyphus*, *Andinobates geminisae*, *Oophaga vicentei*, *Hyloscirtus colymba* (Pers. obs. J.G. Fig 1). The prevalence of SLS in captive-reared amphibians is a factor that introduces uncertainty for population managers and is a research priority for animal husbandry and welfare of captive *Atelopus* [5].

We conducted two controlled captive-rearing experiments designed to improve our understanding of the occurrence of SLS in captive harlequin frogs of the genus *Atelopus*. In the first experiment, we tested the hypothesis that lack of dietary protein in *Atelopus* tadpoles caused SLS. The hypothesis is from anecdotal observations that increasing animal protein in *Mantella aurantiaca* tadpole diets reduced SLS incidents at the National Zoo (L. Augustine, pers. comm).

We tested three hypotheses in the second experiment. The first hypothesis is reducing quantity of food offered might reduce SLS based on anecdotal observations by JG that *Atelopus* tadpoles from the same clutch that were fed lower food quantities, took longer to metamorphose and had a lower SLS incidence. The second hypothesis is that some unknown nutritional factor in our experimental diet may cause SLS based on reports from the Maryland Zoo that they did not observe any SLS in *Atelopus* tadpoles fed exclusively on a commercial food recommended by the Species Survival Program (K. Barrett, pers. comm). The third hypothesis is that reverse-osmosis (RO) treatment of tap water will reduce SLS incidence based on observations that SLS disappeared from the collection following a transition from well water to reconstituted reverse osmosis-treated water at the PARC Project facility at the Nispero Zoo in El Valle de Anton (HR pers. obs).

## Methods and materials

### Experiment 1: Investigating the influence of dietary protein content on SLS

A clutch of *Atelopus certus* eggs laid on 9 December 2014 hatched on 15 December 2014 at the PARC Project facility in Gamboa, Panama. We took 400 tadpoles at Gosner stage 22, two days after hatching, and split them into 16 groups with 25 tadpoles per tank. We used 16 identical 20-liter tanks set up on a single rack with about 2.5 cm of gravel covering an undergravel filter and air stone. Tanks were filled to a 16l mark (80%) and water temperature was maintained at 22.3–24.1C and lighting was provided on a 12-hour cycle from a shop light on each shelf with 2x T8 10% UVB bulbs. Water was partially (20%) changed with carbon-filtered tap water twice weekly through siphoning, in order to reduce fouling. We randomly assigned four tanks to each of four experimental isocaloric diet treatments that contained 31%, 37%, 42% and 48% crude protein respectively (Table 1). These ranges are based on diets of other surrogate frog species, but are within the potential protein content of periphyton collected at sites where other *Atelopus* species have been encountered in Panama (S1 File). *Atelopus* are specialized grazers adapted to eating periphyton, so we weighed food and mixed it to a paste by adding water. We spread the paste > 1mm thickness on acrylic plates and dried it to a film. Each day we replaced two feeding plates in the front and rear of each tank. Initially we offered 0.38 g of food per tank but this led to excessive water fouling. Therefore, we reduced the food offering to 0.24 g per tank after the first 2 weeks. When tadpoles developed hind limbs, we place floating polystyrene slices in the tanks to facilitate froglet emergence. For each tank we recorded 1) proportion of ‘spindly leg’ syndrome by classifying each tadpole into ‘spindly’ or ‘healthy’ [2], 2) mortality or survival through metamorphosis (Gosner stage 47), 3) number of days to metamorphosis of each surviving tadpole. Commercial brand names for equipment and diets used throughout this paper are detailed in S2 File. The Smithsonian Tropical Research Institute IACUC approved this project under proposal #2014–0901.

**Table 1:** Nutritional analysis of all tadpole diets in experiments 1 and 2. Experiment 1 provided isocaloric diets with different crude protein levels. Experiment 2 compared an experimental tadpole diet with a commercially available food. LOQ = undetectable amounts, nd = no measurement provided.

Analysis		Experiment 1				Experiment 2		Unit
		CP31	CP37	CP42	CP48	Expt	Comm	
**Proximates**	Ash	6.4	6.5	6.6	7	10.8	11.1	%
	Crude Fiber	2.2	2.3	2.1	1.6	1.9	2.7	%
	Fat	9.2	9.5	8.6	8	8.1	4.9	%
	Moisture	7.6	7.6	8.6	6.7	7.8	4.7	%
	Protein	31.3	36.6	42.1	48.1	40.2	59.6	%
**Vitamins**	Vitamin B1	23	21.1	20.1	19.1	22.7	10.1	mg/kg
	Vitamin B2	36.5	36.2	37.6	35.1	32.6	17.2	mg/kg
	Vitamin D3	6700	6750	5270	5210	4570	LOQ	IU/kg
	Vitamin A	35700	36500	36300	35300	45000	LOQ	IU/kg
	Vitamin E	393	398	404	405	417	LOQ	mg/kg
	Pantothenic acid	105	107	88.4	108	66.1	16.7	mg/kg
	K3	4.87	5.95	3.87	4.06	8.04	1.55	mg/kg
	Niacin	261	257	256	265	347	161	mg/kg
	Choline	1180	1830	2210	2490	3080	1660	mg/kg
	B6	26.3	nd	23.6	24.3	26.9	4.36	mg/kg
	B12	0.027	0.028	0.028	0.026	0.098	0.613	mg/kg
	Folic acid	7.01	13.4	14.5	20.4	9.2	18.1	mg/kg
	B1 Thiamin	18.1	16.6	15.9	15	nd	nd	mg/kg
	Vitamin C	209	179	178	183	1590	17	mg/kg
**Minerals**	Copper	34.2	13.9	14.5	15.7	20.5	21.3	mg/kg
	Calcium	1.33	1.21	1.16	1.23	1.94	1.2	%
	Magnesium	0.15	0.145	0.136	0.135	0.2	0.317	%
	Manganese	62.3	70.9	67.1	68.1	73.3	33.8	mg/kg
	Iron	260	240	245	255	692	708	mg/kg
	Potassium	0.662	0.686	0.68	0.702	0.701	1.55	%
	Sodium	0.359	0.472	0.587	0.716	0.675	1.68	%
	Zinc	108	99.6	101	108	104	37	mg/kg
	Phosphorus	0.786	0.801	0.782	0.802	1.1	0.979	%
	Selenium	0.77	0.74	0.81	0.79	nd	nd	mg/kg
**Essential Fatty Acids**	Linoleic	1.15	1.27	1.298	1.466	0.554	0.435	%
	Linolenic	0.181	0.203	0.207	0.239	0.108	0.057	%
	Arachidonic	0.027	0.026	0.022	0.019	0.066	0.009	%
	Eicosapentaenoic	0.444	0.441	0.322	0.257	0.501	0.068	%
	Docosahexaenoic	0.456	0.484	0.372	0.295	0.53	0.06	%
**Amino Acids**	Aspartic acid	2.46	3.27	3.98	4.38	3.42	5.61	%
	Serine	1.28	1.65	1.95	2.08	1.61	2.65	%
	Glutamic acid	4.67	5.65	6.49	6.93	5.34	7.88	%
	Glycine	1.97	2.28	2.5	2.54	2.65	2.82	%
	Histidine	0.62	0.82	0.95	0.99	0.85	1.06	%
	Arginine	1.92	2.48	2.98	3.27	2.32	3.54	%
	Threonine	1.07	1.32	1.55	1.64	1.61	2.76	%
	Alanine	1.52	1.82	2.07	2.16	2.12	3.93	%
	Proline	1.75	1.95	2.14	2.223	2	2.14	%
	Cysteine -Total	0.31	0.31	0.35	0.35	nd	nd	%
	Tryosine	0.84	1.11	1.31	1.4	1.17	2.31	%
	Valine	1.23	1.56	1.86	1.99	1.66	3.35	%
	Methionine -Total	0.81	0.81	0.77	0.71	1.16	1.45	%
	Lysine	1.55	2.08	2.5	2.77	2.72	3.29	%
	Isoleucine	1.05	1.37	1.65	1.8	1.46	3.04	%
	Leucine	1.94	2.52	3.01	3.23	2.6	4.77	%
	Tryptophan	0.31	0.35	0.39	0.45	nd	nd	%
	Phenylalanine	1.18	1.54	1.86	2.04	1.48	2.65	%
	Cysteine	0.36	0.4	0.45	0.48	0.36	0.47	%
	Methionine	0.86	0.8	0.78	0.79	nd	nd	%

### Experiment 2: Investigating water composition, ration and diet type on SLS

A clutch of *Atelopus glyphus* eggs hatched on Nov 27, 2016 at the Panama Amphibian Rescue and Conservation Project in Gamboa Panama. Within 3 days of hatching (Gosner stage 22), we took 720 tadpoles and split them into 36 groups with 20 tadpoles per tank. We used a completely randomized design to examine the effects of ration, diet type and water composition using tadpoles as our experimental units (Table 2). We established four replicate tanks per treatment. We offered a custom-made experimental diet designed to meet recommended nutritional requirements for amphibians at full, half and quarter rations to each treatment group. We tested for differences between the experimental diet and the commercial diet type at the half-ration level (Table 2). We provide full nutrient composition analyses conducted in the same laboratory using the same experimental batches for both diet types in Table 1. Our final treatment included the commercial diet and water supplemented with a vitamin b complex in the following ratios: Vitamin B1 10mg/ml, Vitamin B2 2mg/ml, Vitamin B6 2 mg/ml, Vitamin B12 50μg/, D-panthenol 2.6mg/, Nicotinamide 50mg/ml. To compare the effect of water composition we compared municipal tap water provided by the Gamboa Municipality with reconstituted RO water. We prepared RO water in 90L reservoirs using a non-commercial 100G gallon per day Reverse Osmosis System and reconstituted the RO water by dissolving 0.0395g calcium chloride, 0.0465g magnesium sulfate, 0.0358g potassium bicarbonate and 0.0298g sodium bicarbonate per liter of RO water. We measured various water composition parameters for tap water, RO water and reconstituted RO using portable colorimeter according to the manufacturer specifications (Table 3), a single sample of tap water and RO water was mailed to Triton Labs, Germany for an elemental water analysis (Table 4)

**Table 2:** Experimental design of experiment 2 showing how tadpoles (experimental units) were replicated in 36 tanks (blocks) to examine the effects of ration, diet type and water composition on SLS syndrome prevalence in *Atelopus* tadpoles.

RATION, DIET	Reverse osmosis water				Tap water			
Full ration, Experimental	20	20	20	20	20	20	20	20
Half ration, Experimental	20	20	20	20	20	20	20	20
Quarter ration, Experimental	20	20	20	20	20	20	20	20
Half ration, Commercial	20	20	20	20	20	20	20	20
Half ration, Commercial + vitB complex	20	20	20	20				

**Table 3:** Water quality and compositional analysis of water used to supply the *A*. *glyphus* experiment using a colorimeter (March 5–14 2017), or a commercial water quality probe denoted by *. Calcium was evaluated using a Calcium Combination Ion Selective Electrode (ISE)**.

Hach #		Tap	RO	RO + salts
	Conductivity μS/cm*	486.5	83.23	277.2
	Tot Dissolved Solids mg/L*	316.55	53.3	183.3
	SAL ppt*	0.23	0.04	0.13
	pH*	6.79	5.78	6.87
8021	Free Chlorine mg/L	0.21	0.3	0.12
8167	Total Chlorine mg/L	0.05	0.17	0.07
	Ca Hardness mg/L**	26.54	24.14	59.16
8048	Phosphorous, reactive mg/L	0.14	0.04	0.06
8051	Sulfate mg/L	16	0	29
8171	Nitrate mg/L (MR)	0.3	0.4	0.3
8507	Nitrite mg/L (LR)	0	0	0
	Ammonia mg/L*	0.02	0.03	0
	Ammonium mg/L*	0.82	0.2	1
8012	Aluminum mg/L	0.006	0.016	0.003
8027	Cyanide mg/L	0.03	0.03	0.05
8506	Free Copper mg/L	0.08	0	0.04
8008	Total Iron mg/L	0.17	0.02	0.03
8034	Manganese mg/L (HR)	0.1	0.2	0.2
8169	Molybdenum, Molybdate mg/L (LR)	0	0	0.02
8009	Zinc mg/L	0.2	0.13	0.1

**Table 4:** Elemental analysis of water used to supply the *A*. *glyphus* experiment (Sample taken March 7, 2017) Reconstituted RO values were not measured, but were calculated for comparison based on the reconstitution recipe.

		Tap water	RO	Reconstituted RO	Units
Unwanted Heavy Metals	**Hg**	0	0	0	μg/l
	**Se**	0	0	0	μg/l
	**Cd**	0	0	0	μg/l
	**Sn**	0	0	0	μg/l
	**Sb**	0	0	0	μg/l
	**As**	0	0	0	μg/l
	**Al**	79	2.91	2.91	μg/l
	**Pb**	0	0	0	μg/l
	**Ti**	0	0	0	μg/l
	**Cu**	51	14	14	μg/l
	**La**	0	0	0	μg/l
	**Sc**	0	0	0	μg/l
	**W**	0	0	0	μg/l
Macro-Elements	**Na**	48	8.57	16.61	mg/l
	**Ca**	22	17	31.2	mg/l
	**Mg**	18	17	26.76	mg/l
	**K**	0	8.84	22.8	mg/l
	**Br**	1.76	0.56	0.56	mg/l
	**B**	0.09	0.06	0.06	mg/l
	**Sr**	0.17	0.06	0.06	mg/l
	**S**	12	16	16	mg/l
Li-Group	**Li**	0	0	0	μg/l
	**Ni**	0	0	0	μg/l
	**Mo**	0	0	0	μg/l
I-Group	**V**	0	0	0	μg/l
	**Zn**	33	0.66	0.66	μg/l
	**Mn**	3.88	0	0	μg/l
	**I**	7.7	0	0	μg/l
Fe-Group	**Cr**	0	0	0	μg/l
	**Co**	0	0	0	μg/l
	**Fe**	18	0	0	μg/l
Ba-Group	**Ba**	38	0.55	0.55	μg/l
	**Be**	0	0	0	μg/l
Si-Group	**Si**	12891	201	201	μg/l
Nutrient-Group	**P**	0	0	0	μg/l
	**PO4**	0	0	0	mg/l

We used 36 identical tanks filled to a 26-liter mark without any gravel or lighting other than ambient fluorescent overhead shop lights in the laboratory. We placed a 5cm PVC “T” fitting at the bottom of each tank as cover for the tadpoles. We filtered water in each tank using a small sponge filter with air stone in each tank. We added a supplemental air stone to each tank to improve aeration and circulation of water and maintained the temperature at 22.3–22.5°C. We partially (30%) changed the water three times per week through siphoning to prevent water fouling. Weighed food was and mixed with water to a paste then spread to about 1mm thickness on acrylic plates scoured with sandpaper to allow better adhesion. Initially we offered 0.88g as the full ration but reduced this to 0.44g after two weeks due to excessive water fouling, adjusting the half and quarter rations similarly. Once per month, immediately prior to water changes we measured temperature, pH, dissolved oxygen, total dissolved solids, ammonium and ammonia in every tank using a commercial water quality probe. When tadpoles developed hind limbs, we placed floating polystyrene slices in the tanks to facilitate froglet emergence. For each tank we recorded: 1) proportion of SLS by classifying each tadpole into ‘spindly’ or ‘healthy’; [2]; 2) mortality or survival through metamorphosis (Gosner stage 47); 3) number of days to metamorphosis of each surviving tadpole. Any animals displaying spindly leg syndrome were humanely euthanized by immersion in saturated, buffered MS222 as advocated in AMVA humane euthanasia guidelines. The Smithsonian Tropical Research Institute IACUC approved this project under proposal #2014-0901-2017-2-A1.

### Statistical analysis

We visualized data by plotting mean values per treatment +/- standard error. In order to maximize the power to detect statistical differences we defined individual tadpoles as experimental units. Using individual binary response variables such as spindly/healthy required the use of a generalized linear mixed model (GLMM) to analyze data. A GLMM permitted binary data responses and allowed us to incorporate blocking factors such as tank as random covariates [22]. We conducted all analyses using the Package ‘lme4’ in R [23]. In each model, we examined the main effects such as protein, ration, water composition and diet type. Random covariates accounted for variation introduced from other factors including tank and differential tadpole survival in each tank as a proxy indicator for unintended density effects. All relevant data are within the paper and its supporting Information files. Raw experimental data are available in S3 File.

## Results

### Experiment 1: Investigating the influence of dietary protein content on SLS

**Fig 2:**
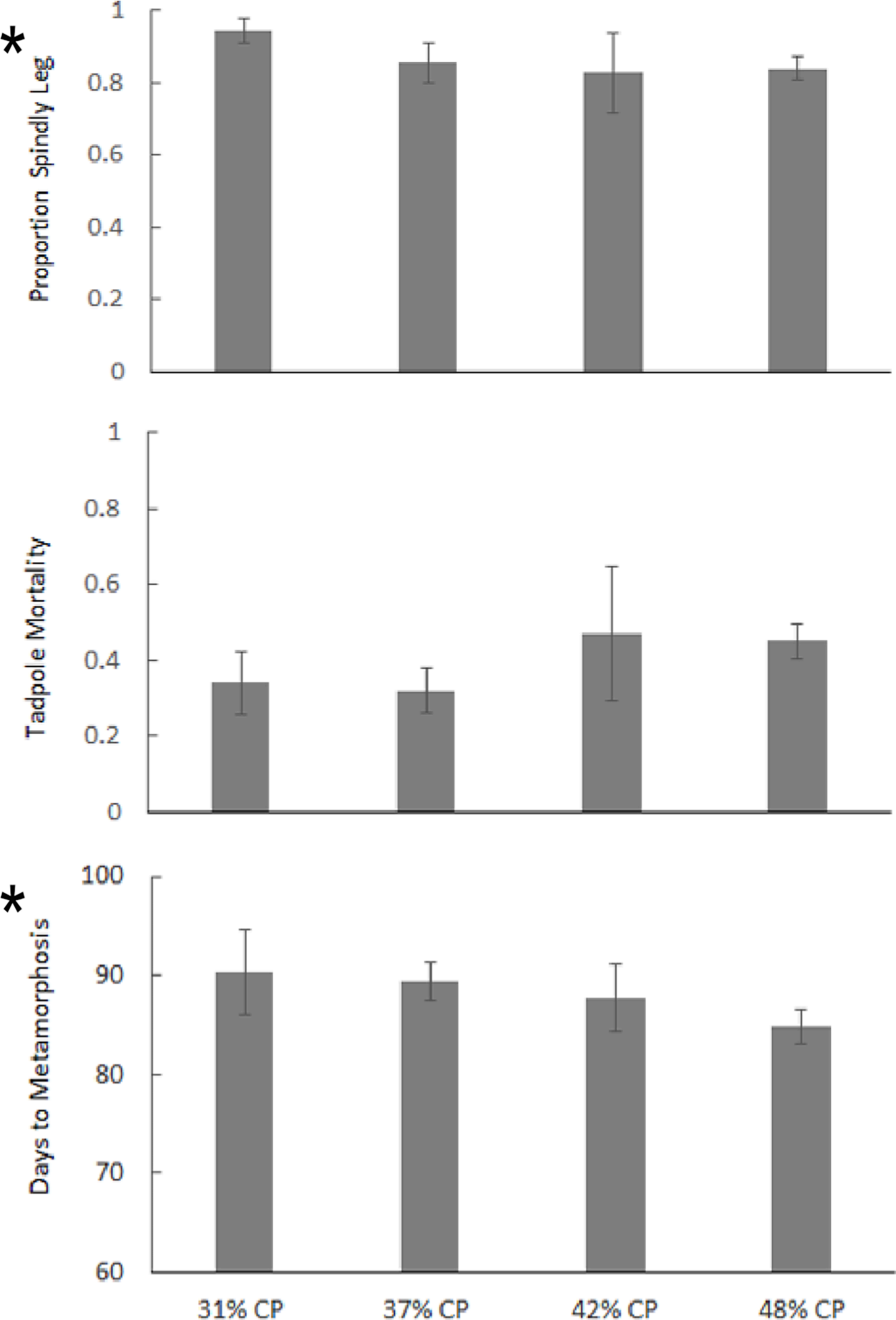
Effects of dietary protein on A) proportion of SLS cases, B) tadpole mortality and C) average number of days to metamorphosis in an experiment with 400 *Atelopus certus* tadpoles. Bars for each treatment represent mean values from 25 tadpoles in each of 4 tanks per treatment +/- SEM, GLMM model provided in (Table 5).

All protein treatments produced more than 80% spindly-leg metamorphs and experienced average mortality rates of 30–50% (Fig 2). A slight, but statistically significant trend indicated that higher protein diets let to metamorphosis a few days earlier than lower protein diets and was associated with marginally lower percentages of SLS (Fig 2, Table 5). However, the overwhelming proportion of SLS metamorphs produced by all treatment groups in this experiment leads us to conclude that dietary protein levels are not the primary causative factor associated with SLS. The high quantities of B vitamins in each experimental diet (Table 1), and the provision of UVB lights for all tanks leading us to conclude that neither UVB nor thiamine were likely limiting factors causing the observed SLS.

**Table 5:** Generalized Linear Mixed Model analysis for Experiment 1 examining the effects of dietary protein content on 1) SLS, 2) mortality and 3) days to metamorphosis in 400 *Atelopus certus* tadpoles that were fed isocaloric diets in four treatments of 28, 34, 40 and 46% protein, replicated in four tanks per treatment and 25 tadpoles in each tank, means shown in Fig 2. Block effects of tank and density-dependent effects resulting from differential mortality between tanks were accounted for in the model as covariates where applicable.

**Model 1: SLS ~ Protein +(1|Tank) +(1|Mortality Rate)** AIC = 201						
		Numerator df	Denominator df	F-ratio	p-value	Covariance
Fixed Effects	**Protein**	**1**	**221**	**5.76**	**0.017**	
Covariates	Tank					>0.001
	Mortality Rate					0.024
	Error					0.131
**Model 2: Mortality ~ Protein +(1|Tank)** AIC = 525						
		Numerator df	Denominator df	F-ratio	p-value	Covariance
Fixed Effects	Protein	1	372	0.043	0.836	
Covariates	Tank					>0.001
	Error					0.235
**Model 3: Days to Metamorphosis ~ Protein +(1|Tank) +(1|Mortality Rate)** AIC = 3193						
		Numerator df	Denominator df	F-ratio	p-value	Covariance
Fixed Effects	**Protein**	**1**	**221**	**4.11**	**0.04**	
Covariates	Tank					0.003
	Mortality Rate					338.1
	Error					189.9

### Experiment 2: Investigating water composition, ration and diet type on SLS

**Fig 3:**
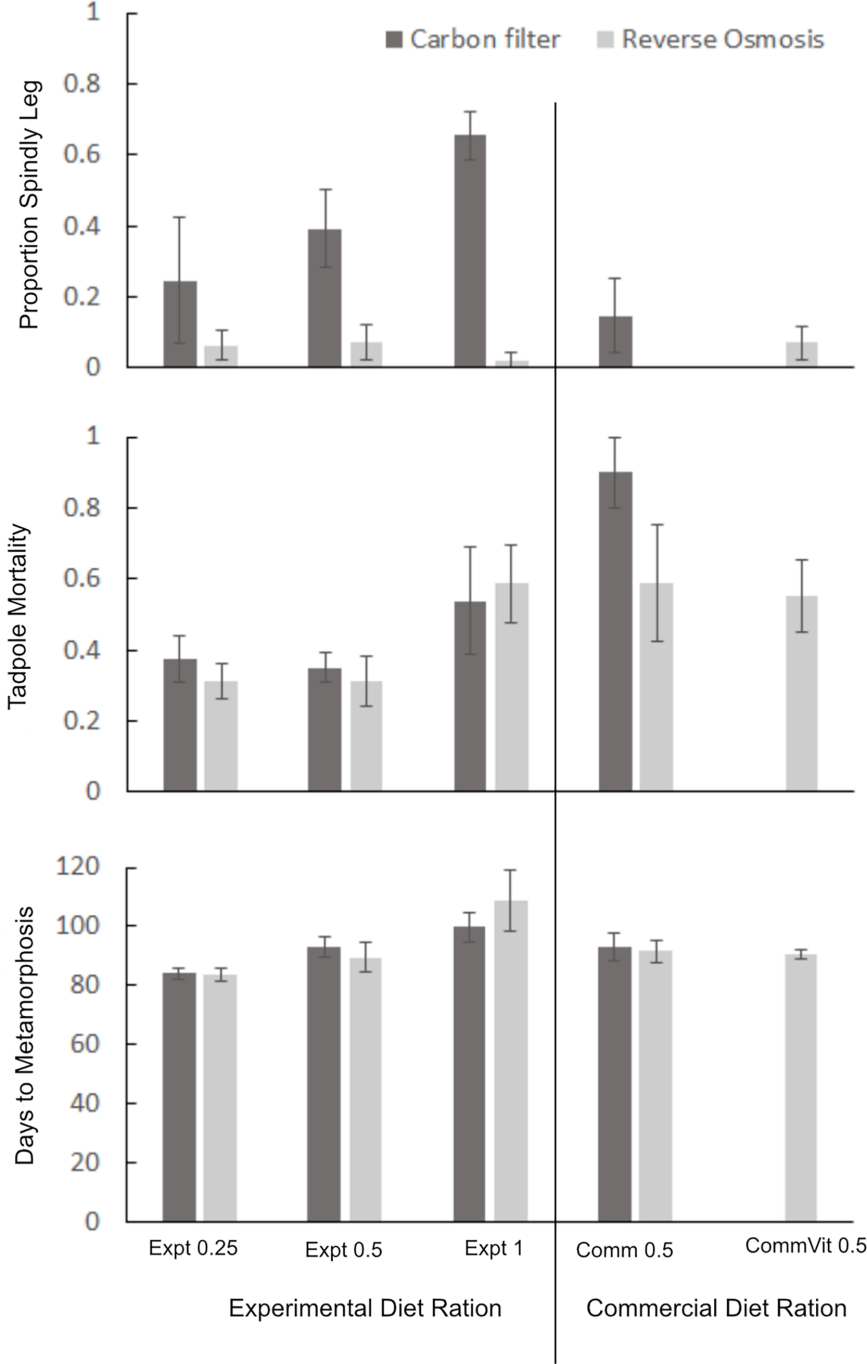
Effects of diet type, ration and water source on A) proportion of SLS cases, B) tadpole mortality and C) average number of days to metamorphosis in *Atelopus glyphus* tadpoles. Bars for each treatment represent mean values from 4 tanks +/- SEM. 20 tadpoles were placed in each tank at the beginning of the experiment. Proportion of SLS for commercial 0.5, RO water is not presented because 100% tadpole mortality in some tanks led to missing replicates.

Diet type did not significantly affect the prevalence of SLS, but both water source and ration significantly influenced spindly leg incidence with probabilities > 0.001 (Table 6). Regardless of ration, the provisioning of reconstituted RO water drastically reduced the incidence of SLS (Fig 3). Despite the severe reduction in SLS cases with reconstituted RO water, they were not completely eliminated. A closer examination of the RO water data (Table 3) indicates that the RO process removed about 84% of the total dissolved solids in the tap water so it is likely that a small proportion of all the dissolved solids remained even post RO treatment. The regular tap water treatments had consistently higher incidence of spindly leg than reconstituted RO treatments but the proportion of SLS in tap water treatments was correlated with ration. Tadpoles receiving a full ration had about 70% SLS and dropped to about 25% spindly in the quarter ration treatments (Fig 3). It is unclear which water composition parameters were responsible for the dramatic differences between RO and tap water. A single measurement of the starting ratio of Phosphate to total Calcium hardness in tap water was 1:189, while in the reconstituted RO it was 1:986. Starting concentrations of total dissolved solids, chlorine, phosphates, and iron were substantially higher in tap water compared to RO, while RO had higher sulfate concentrations (Table 3). Multiple measurements of a more limited set of parameters in the actual tanks revealed that dissolved solids, ammonia and ammonium were consistently higher in RO treatments than tap water (Table 7). This pattern was not evident in the starting concentrations of the water provided (Tables 3 & 4), indicating that some differential biological processes may be occurring in the different water treatment groups. Furthermore, post-metamorph survivorship rates in RO treatments were double those of regular tap water (Table 8).

**Table 6:** Linear Mixed Models examining the effects of ration, water and diet on 1) SLS, 2) mortality and 3) days to metamorphois in 720 *Atelopus glyphus* tadpoles reared in 36 tanks with 20 tadpoles per tank and 4 tanks per treatment. Block effects of tank and density-dependent effects resulting from differential mortality between tanks were accounted for in the model as covariates where applicable.

**Model 1: SLS ~ Ration + Water + Diet +(1|Tank) +(1|Mortality Rate)** AIC = 297						
		Numerator df	Denominator df	F-ratio	p-value	Covariance
Fixed Effects	**Ration**	**1**	**369**	**13.3**	**>0.001**	
	**Water**	**1**	**369**	**65.8**	**>0.001**	
	Diet	2	369	2.1	0.121	
Covariates	Tank					>0.001
	Mortality Rate					>0.001
	Error					0.121
**Model 2: Mortality ~ Ration + Water + Diet +(1|Tank)** AIC = 1023						
		Numerator df	Denominator df	F-ratio	p-value	Covariance
Fixed Effects	**Ration**	**1**	**714**	**13.1**	**>0.001**	
	Water	1	714	1.0	0.315	
	**Diet**	**2**	**714**	**11.1**	**>0.001**	
Covariates	Tank					>0.001
	Error					0.235
**Model 3: Days to Metamorphosis ~ Ration + Water + Diet +(1|Tank) +(1|Mortality Rate)** AIC = 3193						
		Numerator df	Denominator df	F-ratio	p-value	Covariance
Fixed Effects	**Ration**	**1**	**369**	**37.6**	**>0.001**	
	Water	1	369	0.4	0.505	
	Diet	2	369	1.3	0.248	
Covariates	Tank					0.006
	Mortality Rate					33.59
	Error					297.6

**Table 7:** Summary of water quality variables by treatment using a water quality probe. Means for each treatment were determined measuring all 36 tanks on 4 separate sampling dates immediately prior to water changes. Comm = commercial diet, Expt = experimental diet, CommVit = Commercial diet with vitamin B complex supplement added to the water. DO = Dissolved oxygen, TDS = Total Dissolved solids.

Diet	Ration	Water	Temp	pH	DO%	TDS	NH4 mg/L	NH3 mg/L
**Comm**	**0.5**	**Tap**	22.4	7.26	86	293	0.24	0.000
**Expt**	**0.25**	**Tap**	22.3	7.48	88	289	0.22	0.000
**Expt**	**0.5**	**Tap**	22.4	7.42	88	286	0.19	0.000
**Expt**	**1**	**Tap**	22.3	7.38	86	292	0.33	0.003
**Comm**	**0.5**	**RO**	22.5	7.63	87	400	0.92	0.017
**CommVit**	**0.5**	**RO**	22.3	7.45	85	399	0.83	0.013
**Expt**	**0.25**	**RO**	22.4	7.69	87	388	1.37	0.033
**Expt**	**0.5**	**RO**	22.4	7.69	87	387	1.07	0.024
**Expt**	**1**	**RO**	22.5	7.64	87	392	1.02	0.021

**Table 8:** Summary of *Atelopus glyphus* post-metamorph survivorship at 376 days.

	Number emerged healthy	Number emerged spindly	% Spindly	Number of Survivors 6 Dec	Survival rate healthy post-metamorphs
Total frogs RO	204	10	9.6	116	56.9
Total frogs Tap water	104	60	57.7	28	26.9

Diet type and ration significantly affected tadpole mortality but type of water did not (Table 6). Both commercial diets and the full ration experimental diets were associated with reduced tadpole survival (Table 6, Fig 3) and provisioning of supplementary vitamins to the water of tadpoles fed the commercial diet had no effect on survival (Fig 3). Ration was the only experimental factor that influenced the number of days to metamorphosis. We designed the experiment anticipating that limited food availability would be a treatment that would be associated with slower growth rates. Counter to our expectations, however, tadpoles receiving a full ration took on average 15–20 days longer to metamorphose than tadpoles receiving a quarter ration (Fig 3). This observation, combined with the higher mortality rates observed in full ration diets leads us to conclude food quantity was not a limiting factor for any of the diet treatments. In fact, it is likely that all ration treatments were overfeeding the tadpoles. Despite the three times per week water changes, it seems likely that water fouling associated with the overfeeding is likely to be responsible for the slower development and higher mortality rates observed in higher ration treatments (See Table 7 for water quality data immediately prior to water changes).

## Discussion

### Experiment 1: Investigating the influence of dietary protein content on SLS

Similar to other tadpole experiments, we found that tadpoles with higher protein diets metamorphosed sooner. Other studies have noted that the optimum protein levels in bullfrog and natterjack toad tadpole diets are 44–46% [24,25], while extremely reduced dietary protein in tadpoles impairs growth rates and disease resistance [26]. Despite a relative abundance of studies of dietary protein on tadpole development, none report any relationship to diet and poor limb development [25–28]. The lack of any wild-type dietary baselines for *Atelopus* species is a limiting factor, forcing us to use surrogate species that may differ substantially in their biological requirements. The preliminary data on nutritional composition of food available to *Atelopus* tadpoles (S1 File), shed more light on the difficulties associated with this task, than the actual nutritional composition of the food. The high prevalence of SLS across all protein treatments in this experiment and the lack of SLS encountered in other dietary protein studies lead us to conclude that dietary protein is not a causal factor associated with SLS. Two commonly cited limiting factors involved in the endocrine regulation of calcium are the provisioning of UVB light, and vitamin D3 content of the diet [29], but these factors were not limiting in this experiment and UVB was provisioned at 2.6–3.5 UVI, and vitamin D3 levels were 6 times the recommended concentrations [1]. The Ca:P ratio of these tadpole diets was about 1.5:1, exceeding the recommended minimum ratio of 1:1 for adult amphibians [9]. Recommended Ca:P ratios for tadpole diets have not been determined but may be a less-relevant factor, given tadpoles’ ability to absorb calcium from their environment [12].

### Experiment 2: Investigating water composition, ration and diet type on SLS

The clearest pattern from this experiment was that provision of reconstituted RO water drastically reduced the occurrence of SLS, and we can say with some confidence that differences in water composition were responsible for the observed differences. Given our knowledge of the importance of calcium metabolism in amphibian metamorphosis [12], a more detailed experimental examination of Ca:P ratios in *Atelopus* tadpole water seems warranted. According to the U.S. Geological Survey, soft waters are less than 60 mg/l hardness, while very hard water exceeds 251 mg/l on the water hardness scale [30]. Our Gamboa experimental water is on the lower end of the scale with Ca hardness values of 26 mg/l in tap water 56 mg/l in reconstituted water (Table 3). One other study noted scoliosis and crooked limb development in tadpoles reared in water with a hardness <4mg/l [18] but it is not clear if SLS was observed. A second study observed hind limb deformities connected with poor neuron development that were induced in deionized water that were ameliorated by the addition of CaCl_2_ [19]. Based on these similar, independent observations, it seems likely that a lack of dissolved calcium in the water is a potential limiting factor in this system.

A second clear pattern within tap water treatments is that the prevalence of SLS was aggravated by overfeeding. Traditional indicators of poor water quality such as ammonia were actually higher in the reconstituted RO treatments (Table 7, Kruskall Wallace Test 1df, Chi square 27, p <0.001). The fact that the tanks with the poorest water quality indicators of organic matter decomposition had the lowest spindly leg incidence indicates that ammonia release was not the likely factor causing SLS (Table 3). It is possible, however, that some other soluble factor associated with surplus food decomposition in the tap water could be cumulatively adding undesirable elements to the water in both the RO and the tap-water treatments. The more advantageous starting water composition provided by the RO treatment mitigated the effects somewhat in that treatment. In light of these results, the combination of tap water and overfeeding associated with all treatments in experiment 1 may explain the high spindly leg ratios consistently observed in that experiment.

The dietary analyses showed that the experimental diets compared favorably to the recommended amphibian nutrition intake for post-metamorphic amphibians (Table 3,[1]). The diet type did not significantly affect SLS prevalence but it did significantly affect mortality rates, with the highest mortality rates observed in the commercial diet treatments, and this may be connected to vitamin deficiencies noted in the nutritional analysis of that diet.

Overall, the results of these experiments lead us to conclude that spindly leg syndrome is directly related to the water composition in which the tadpoles are raised, rather than the diet. Further experiments should experimentally focus on the addition or removal of specific compounds to water that can reduce the incidence of spindly leg.

## Supporting information

S1 File Periphyton collection from historical *Atelopus* habitat. Details of attempted collection and nutritional analysis of diatoms and algae growing in *Atelopus* habitat.
(DOCX)

S2 File Brand information. Account of brands, makes and models used in this experiment.
(DOCX)

S3 File Experimental data (excel file). Tab 1: Raw results data from experiment 1. Tab 2: Raw results data from experiment 2.
(XLSX)
