# Supplementary material for: The relationship between spindly leg syndrome incidence and water composition, overfeeding, and diet in newly metamorphosed harlequin frogs (Atelopus spp.)
Source: PLoS One. 2018 Oct 16;13(10):e0204314. doi: 10.1371/journal.pone.0204314 (PMC6191089; doi:10.1371/journal.pone.0204314)
Supplement: S1 File — Details of attempted collection and nutritional analysis of diatoms and algae growing in Atelopus habitat. (DOCX) [file pone.0204314.s001.docx]

# S1: Periphyton collection from historical *Atelopus* habitat

# Methods

We sampled periphyton from three historical or current *Atelopus* streams in 2015. Two sites in El Valle de Anton (La India Dormida, and Hotel Campestre) were formerly occupied by *Atelopus zeteki* (1) and the third site near Colon (Sierra Llorona) is currently occupied by *Atelopus limosus* (R.I. pers comm). Fist-size or larger rocks were collected from riffles and periphyton was gently scrubbed from their surface with a nylon-bristled brush into a bucket. We filtered the water through a WaterMark® Simple Plankton Net (50 micron) to concentrate the periphyton. This fine mesh size was required due to the small size of diatoms known from *Atelopus* habitat (2). Several collections ranging from 3-5 hours of collective effort per site were made in order to amass a sample large enough to conduct basic nutrient analyses >3g dry weight. Samples were lyophilized then exported to the Smithsonian National Zoo nutrition laboratories in Washington DC (Collection permit number SE/A-92-14, export permit number SEX/P-7-16). Nitrogen (N) was measured using an elemental gas analyzer (Model 2400, Perkin Elmer, Norwalk CT). We validated this method against the macro Kjeldahl procedure with nitrogen recovery around 98-99%. Total crude protein was calculated as 6.25 X N (3). Ash was determined by placing dried samples in a muffle furnace at 550°C for 8 hours. The samples had a high sand content resulting from scrubbing the rocks, therefore ash values represent total mineral for both sand and organisms in sample. The samples were unstable in perchloric acid digestion and no further mineral analysis was conducted. The organic matter content was determined by subtracting the ash weight from the starting dry sample weight with protein expressed as a percent of the total organic matter. We did run the samples for fat with petroleum ether extraction, but it was undetectable most likely due to the high sand content.

# Results

We found that the crude protein levels of periphyton available for *Atelopus* at three sites ranged widely from 23-50 % of the organic matter with a mean value of 32% (Table 5). We could not provide any further proximate analyses due to the high sand content of the samples. A visual inspection of 20 microscope slides prepared from the samples showed that diatoms, detritus, sand and filamentous algae dominated the samples in that approximate order of importance, but we made no further effort to quantify these observations.

**Table S1:** Crude protein estimates of periphyton available to *Atelopus* tadpoles scrubbed from stream boulders in *Atelopus* habitat

| **Stream** | N | Mean Adjusted Crude Protein | Range |
| --- | --- | --- | --- |
| India Dormida | 11 | 44% | 41-50% |
| Hotel Campestre | 11 | 26% | 23-28% |
| Sierra Llorona | 3 | 27% | 23-32% |
| **Mean** |  | **32%** | **23-50%** |

# Discussion

In the wild, periphyton available to *Atelopus zeteki* tadpoles is composed of preferentially-grazed, larger diatom species including *Amphipleura lindheimerii* and *Gyrosigma acuminatum* (4). The nutrient profiles of these algae are unknown and we have a poor understanding of nutritional baselines when developing artificial diets for ex-situ assurance colonies. The range of 23-50% protein observed in wild periphyton indicated that the ranges of protein content in the diets we tested were appropriate Sera® Micron (mixture of spirulina and krill) or Hikari® Algae Wafers (mixture of fishmeal, wheat flour and seaweed) are the recommended artificial diets for *Atelopus* tadpoles (5), and they vary widely in protein content with 52% and 33% protein respectively. The algae wafers approximate the mean available protein in wild diet types (32%), but is lower than the optimum protein content of 44% observed for American bullfrogs *Rana catesbeiana* (6). The 52% protein content of Sera® Micron, however, exceeds the 50% upper limit of the available range we observed in wild periphyton.

We could not conduct further proximate analyses because the high sand content interfered with our ability to evaluate fat in such small samples, and the samples would explode during the perchloric acid digestion. Future workers will want to focus on better ways to remove the sand fraction from the sample prior to analysis, or develop other collection methods. Ideally the samples would be collected from multiple sites within a single species’ range.

# References

1. NMNH. Smithsonian National Museum of Natural History Amphibians and Reptiles Collections Database [Internet]. https://collections.nmnh.si.edu/search/herps/. 2017 [cited 2017 Nov 17].

2. Ranvestel AW, Lips KR, Pringle CM, Whiles MR, Bixby RJ. Neotropical tadpoles influence stream benthos: evidence for the ecological consequences of decline in amphibian populations. Freshw Biol 2004;49(3):274–85. doi: 10.1111/j.1365-2427.2004.01184.x

3. Jones DB. Factors for converting percentages of nitrogen in foods and feeds into percentages of proteins. US Department of Agriculture Washington, DC; 1941.

4. Connelly S, Pringle CM, Bixby RJ, Brenes R, Whiles MR, Lips KR, et al. Changes in stream primary producer communities resulting from large-scale catastrophic amphibian declines: Can small-scale experiments predict effects of tadpole loss? Ecosystems. 2008;11(8):1262–76.

5. Poole V. Husbandry Manual Panamanian Golden Frog *Atelopus zeteki*. Vol. 2nd Edn. Baltimore; 2006.

6. Carmona-Osalde C, Olvera-Novoa M., Rodríguez-Serna M, Flores-Nava A. Estimation of the protein requirement for bullfrog (*Rana catesbeiana*) tadpoles, and its effect on metamorphosis ratio. Aquaculture. 1996;141(3–4):223–31.
