# Supplementary material for: The relationship between spindly leg syndrome incidence and water composition, overfeeding, and diet in newly metamorphosed harlequin frogs (Atelopus spp.)
Source: PLoS One. 2018 Oct 16;13(10):e0204314. doi: 10.1371/journal.pone.0204314 (PMC6191089; doi:10.1371/journal.pone.0204314)
Supplement: S2 File — Account of brands, makes and models used in this experiment. (DOCX) [file pone.0204314.s002.docx]

# S2: Brand information

We eliminated brand information from the text of the paper as a measure avoid appearance of conflicts of interests. We provide them here in the interests of experimental repeatability.

# Experiment 1:

- UV Bulbs: T8 ReptiSun® 10.0 UVB bulbs
- Custom isocaloric diets were prepared and nutrient profiles analyzed by the Waltham Center for Pet Nutrition.

# Experiment 2:

- The custom diet was prepared and nutrient profiles analyzed by the Waltham Center for Pet Nutrition.
- The Commercial diet used was Sera® Micron, which is a Spirulina Krill mix recommended by the Golden Frog Species Survival Plan (5).
- Reverse osmosis system: Aqua FX® AF3300C Dolphin Reverse Osmosis System.
- Water quality variables were measured using a Hach® DR 890 portable colorimeter, a YSI Professional plus 13c100785, and a Hach Sension+ 9660C Calcium Combination Ion Selective Electrode (ISE).
- Elemental analyses were conducted by a third party laboratory Triton Labs, Germany.
- The sponge filter was a Hikari® Aquarium Solutions Bacto-Surge filter for 10-gallon tanks.
